# Maternal outcomes associated to psychological and physical intimate partner violence during pregnancy: A cohort study and multivariate analysis

**DOI:** 10.1371/journal.pone.0218255

**Published:** 2019-06-13

**Authors:** Stella Martin-de-las-Heras, Casilda Velasco, Juan de Dios Luna-del-Castillo, Khalid S. Khan

**Affiliations:** 1 Department of Forensic Medicine, University of Granada, Granada, Spain; 2 Department of Nursing and Midwifery, University of Jaen, Jaen, Spain; 3 Department of Biostatistics, University of Granada, Granada, Spain; 4 Women’s Health Research Unit, Queen Mary University of London, London, United Kingdom; Stellenbosch University, SOUTH AFRICA

## Abstract

Intimate partner violence (IPV) is a public health problem that affects millions of women worldwide and can occur during both pregnancy and the perinatal period. We aimed to evaluate if the experience of psychological and physical intimate partner violence (IPV) adversely affects pregnancy outcomes. We established a cohort of 779 consecutive mothers receiving antenatal care including ultrasound and giving birth in 15 public hospitals, drawn using cluster sampling of all obstetric services in Andalusia, Spain (February-June 2010). Trained midwives gathered IPV data using the Index of Spouse Abuse validated in the Spanish language (score ranges: 0–100, higher scores reflect more severe IPV; cut-offs: physical IPV = 10, psychological IPV = 25). Socio-demographic data, including lack of kin support, maternal outcomes, and hospitalization were collected. Multivariate logistic regression estimated adjusted odds ratios (AOR), with 95% confidence intervals (CI), of the relationship between psychological and physical IPV and maternal outcomes, controlling for socio-demographic characteristics. Response rate was 92.2%. Psychological IPV, reported by 21.0% (n = 151), was associated significantly with urinary tract infection (127 (23%) vs 56 (37%); AOR = 1.9; 95%CI = 1.2–3.0), vaginal infection (30 (5%) vs 20 (13%); AOR = 2.4; 95%CI = 1.2–4.7) and spontaneous preterm labour (32 (6%) vs 19 (13%); AOR = 2.2; 95%CI = 1.1–4.5). Physical IPV, reported by 3.6% (n = 26), was associated with antenatal hospitalizations (134 (19%) vs 11 (42%); AOR = 2.6; 95%CI = 1.0–7.1). Lack of kin support was associated with spontaneous preterm labour (AOR = 4.7; 95%CI = 1.7–12.8). Mothers with IPV have higher odds of complications. Obstetricians, gynaecologists and midwives should act as active screeners, particularly of the undervalued psychological IPV, to reduce or remedy its effects.

## Introduction

Violence against women including intimate partner violence (IPV) is a global public health problem and a fundamental human rights breach [1]. Psychological abuse in a current or past intimate relation is increasingly being recognized, over and above physical violence [2,3]. Pregnancy represents a period of particular vulnerability [4], with reported IPV prevalence higher than many common obstetric conditions [5], varying across countries and cultural contexts [4,6–8].

The literature on effects of IPV during pregnancy has conflicting results [9]. Limitations in statistical power due to insufficient sample sizes, and risk of bias due to deficiencies in study methodology are known [10,11]. Variations in operational definitions, both for exposures and outcomes, weaken any associations observed. The lack of consistent, valid and reliable assessments of IPV [12] with many studies focusing solely on physical abuse [13] have left the area of psychological abuse during pregnancy largely ignored. There have been calls for continued and improved investigation particularly as the detrimental consequences of non-physical abuse are under-recognised [14–17].

Based on a review of the literature [9–20], we hypothesized that physical and psychological IPV during pregnancy might contribute to maternal morbidity through an association with obstetric complications (e.g. preeclampsia, gestational diabetes). We evaluated if the experience of psychological or physical IPV captured through validated tools in pregnancy adversely affects maternity outcomes in a cohort study.

## Materials and methods

### Population, sample size and study subjects

A population-based study was designed based on 2009 regional health service statistics for all public hospitals (n = 28) in Andalusia, Spain (number of births = 76,336). A cluster sampling approach was adopted, considering the hospitals as clusters grouped by hospital type (regional = 5; specialized = 10; district = 13). A sample size of 750 women was estimated to provide an accuracy of ±2.5% with 99% confidence for IPV detection, assuming an IPV prevalence of 7.5% (a review of the literature [12] suggested a rate ranging 4–8% in comparable populations) and an intraclass correlation coefficient among the hospitals of 5% [21]. The sample numbers were reached by enrolling 50 women each from 15 hospitals randomly selected to represent the hospital type [8]. A total of 779 women participated in this study. Included were women admitted to obstetrics departments antenatally and giving birth within the study period. Excluded were women with stillbirths, those unable to communicate in the Spanish language, and those with disease or disability preventing collection of the study data (e.g. women with mobility problems limiting access the private room for the interview).

The study was approved by the research ethics committees of all participating hospitals: Hospital Universitario Reina Sofía (26th March, 2009), Hospital Regional Universitario Carlos de Haya (25th June, 2009), Hospital Universitario San Cecilio (27th September, 2010), Hospital Universitario Virgen del Rocío (11th March, 2010), Hospital Juan Ramón Jiménez (19th October, 2009), Hospital Torrecárdenas (20th November 2009), Hospital de Jerez (9th July, 2009), Hospital Virgen Macarena (16th October, 2009), Hospital Universitario de Puerta Real (25th June, 2010), Hospital Virgen de las Nieves (30th October, 2009), Hospital de Baza (17th September, 2010), Hospital Punta de Europa (12th February, 2010), Hospital Universitario de Jaén (28th May, 2010), Hospital Axarquía (17th June, 2010) and Hospital Universitario de Valme (3rd March, 2010). Please, note that in our regions, studies are identified by ethical committees using protocol titles and date of approval; therefore we do not have a reference number for the approval. The form of the consent obtained was always written.

### Data collection procedures

Data were collected during the immediate postpartum period by midwives at each hospital who received specific training for the study. Women were recruited on consecutive days until the sample size per hospital was reached (n = 50), avoiding any day without sampling. Data were gathered in one-to-one interviews in a room other than the ward in which the woman was hospitalized, taking care to ensure that partner was not present. The study was explained with guarantees of strict anonymity and confidentiality of the information collected, including compliance with the national regulation that confidentiality is maintained even from health professionals and the police unless the women consented to sharing information. Women participating signed informed consent. If the women’s responses suggested evidence of IPV, comprehensive information concerning the police, judicial, and social services and resources was given.

### Data collection instruments

#### Socio-demographic questionnaire

Data were collected on items such as age, marital status, schooling history, employment, nationality, cohabitation with partner/family, and the availability of next of kin support (i.e. a relative who could be turned to when needed). A non-committed relationship was considered to be one between individuals who may have casual sex without demanding or expecting the commitment of a formal relationship.

#### Experience of IPV

IPV was defined as physical, sexual, coercion or psychological abuse, and controlling behaviours perpetrated by a current or past intimate relation [3,4] during 12 months before giving birth. It was captured in the immediate post-partum period by Index of Spouse Abuse (ISA), a 30-item instrument measuring the severity and frequency of abuse using weighted items (S1 File provides details of positive cases of IPV were identified) [22]. ISA was designed to measure two different types of abuse: an ISA-P score that represents the severity of physical abuse and an ISA-NP score that represents the severity of nonphysical or psychological abuse. It included assessments of emotional abuse (e.g. my partner screams and yells at me), psychological threats (e.g. my partner becomes very angry if I disagree with his point of view), coercive tactics (e.g. my partner orders me around), and physical (e.g. my partner slaps me around my face and head) and sexual abuse (e. g. my partner makes me perform sex acts that I do not enjoy or like). Item weights were used in scoring the ISA to account for the fact that some of the items represented very serious forms of abuse while others were less serious. ISA score ranged from 0 to 100 points where a low score indicated the relative absence of abuse and the higher scores represented the presence of a greater degree or amount of abuse. Two severity scores were computed, one for physical (ISA-P) abuse and the other for non-physical (ISA-NP) or psychological abuse. Recommended cut-off scores were 10 for physical abuse and 25 for psychological abuse as at these thresholds the sum of false positives and false negatives was minimized. Strict adherence to the scoring procedures has been strongly recommended [22]. The instrument was validated for use in Spanish [23].

#### Maternal outcomes

Outcomes during the pregnancy were anemia (<10.5 g/dL), urinary tract infection, vaginal infections (sexually transmitted infection, candidiasis, bacterial vaginosis, etc.), vaginal bleeding (threatened abortion and antepartum hemorrhage), gestational diabetes (confirmed by glucose tolerance test at 24–28 weeks), spontaneous preterm labour (onset of regular uterine contractions associated with progressive cervical change between viability and 37 completed weeks of gestation), gestational hypertension (>140/90 mmHg), or others (e.g. hyperemesis, hypothyroidism, mental disorders, placental disorders, renal colic and intrauterine growth retardation). All women received routine antenatal care including estimation of gestational age by early ultrasound. Women were asked about attendance to emergency service and hospitalization during pregnancy. This information was verified from the prospectively documented individual health records of pregnancy.

### Statistical analysis

The prevalence rate and 95% confidence interval (CI) were calculated for IPV. Chi-square test was used to compare differences in categorical variables. Multiple logistic regression analysis determined the relation between IPV (psychological, physical and combined) and various maternity outcomes, attendance at the emergency service and antenatal hospitalization. Gestational diabetes, gestational hypertension and chronic hypertension (latter included in “other pathologies”) have been included in the analysis but they were found not to have any cofounding effect within our model. To improve fit and precision of estimation, we dropped these from the final model. The models controlled for age, marital status, educational level, employment status, nationality, cohabitation, and kin support. The attendance to emergency service model and antenatal hospitalization model were also adjusted by obstetric complications. The results were summarised as adjusted odds ratios (AORs) with 95% CIs.

## Results

**Fig 1:**
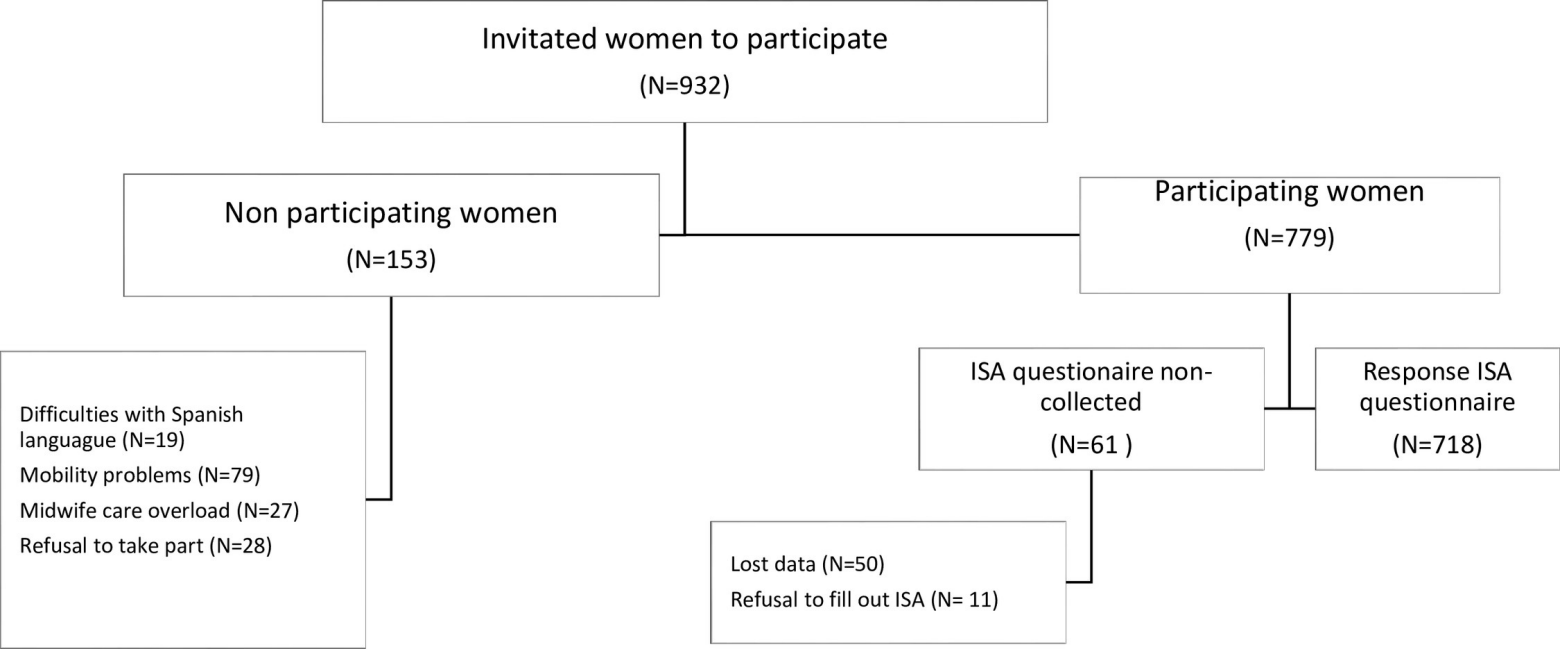
Flow diagram of the participants. ISA: Index of Spouse Abuse.

IPV in pregnancy was reported by 21.3% (n = 153) of the women, including physical and/or psychological IPV, with no duplication of cases. Physical IPV was reported by 3.6% (n = 26) and psychological by 21.0% (n = 151). The prevalence of women experiencing both, physical and psychological IPV, during pregnancy was 3.3% (n = 24). A flow diagram of the participants and the socio-demographic characteristics of the sample were shown in Fig 1 and Table 1, respectively. The response rate was 92.2% and the lost data 4.3%.

**Table 1:** Socio-demographic characteristics of the sample.

	N	Fr (%)	Psychological IPV^a^ N (%)	Physical IPV^a^ N (%)
**Age**^b^ **yrs.**				
<20	26	3.7	12 (46.1)	2 (7.7)
20–24	95	13.6	29 (30.5)	2 (2.1)
25–29	187	26.8	43 (23.0)	10 (5.4)
30–34	260	37.2	39 (15.0)	8 (3.2)
35–39	104	14.9	18 (17.3)	2 (1.9)
≥40	26	3.7	5 (19.2)	2 (7.7)
**Relationship status**				
Married	466	65.1	67 (14.4)	8 (1.7)
Committed	102	14.2	27 (26.5)	5 (4.9)
Non-committed	148	20.7	56 (37.8)	13 (8.8)
**Years of schooling**				
<7	262	36.5	68 (25.9)	11 (4.2)
7–12	350	48.8	72 (20.6)	12 (3.4)
>12	105	14.6	11 (10.5)	3 (2.9)
**Employment status**				
Housewife	159	22.2	42 (26.4)	13 (8.2)
Unemployed	143	19.9	34 (23.8)	6 (4.2)
Employed	402	56.1	69 (17.2)	6 (1.5)
Student	13	1.8	5 (38.5)	1 (7.7)
**Nationality**				
Spanish	652	90.8	131 (20.1)	19 (2.9)
Other	66	9.2	20 (30.3)	7 (10.6)
**Cohabitation**				
Partner	657	91.5	126 (19.2)	20 (3.0)
Other	61	8.5	25 (41.0)	6 (9.8)
**Kin support**				
Yes	680	95.1	133 (19.6)	21 (3.1)
No	35	4.9	17 (48.6)	5 (14.3)

^a^IPV: Intimate partner violence

^b^Mean = 29.9 ± 5.6 yrs

**Fig 2:**
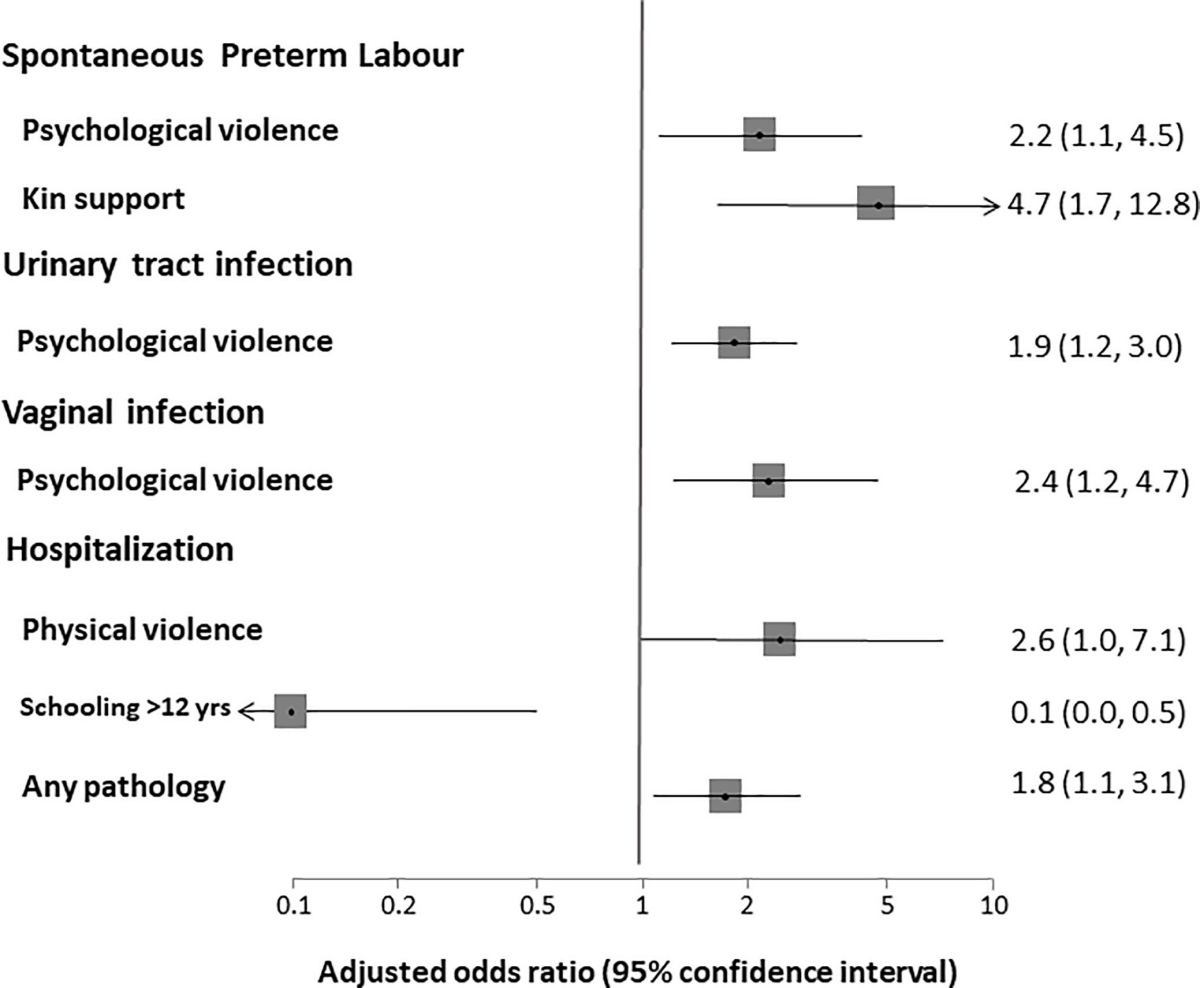
Adjusted odd ratios of multivariate models.

Distribution of the maternal outcomes and statistical associations with psychological or physical IPV during pregnancy are presented in Table 2. Any pathology during pregnancy was presented by 539 women, 124 of them (23%) reported IPV. Anaemia was the most common pathology reported by women during pregnancy (39.3%), followed in frequency by urinary tract infection (25.3%), vaginal bleeding (16.0%), gestational diabetes (8.0%), spontaneous preterm labour (7.1%), vaginal infections (6.9%) and gestational hypertension (6.6%). Psychological IPV was associated with urinary tract infection (AOR = 1.9; 95%CI = 1.2–3.0), vaginal infection (AOR = 2.4; 95%CI = 1.2–4.7) and spontaneous preterm labour (AOR = 2.2; 95%CI = 1.1–4.5) (Fig 2; S1 and S2 Tables). Physical IPV was associated with antenatal hospitalizations (AOR = 2.6; 95% = 1.0–7.1) (Fig 2; S3 Table). The lack of kin support was a risk factor of spontaneous preterm labour (AOR = 4.7; 95% CI = 1.7–12.8). The results for both psychological and physical IPV combined (not reported) were virtually identical to those of the model for psychological IPV.

**Table 2:** Distribution of maternal outcomes.

			Psychological IPV^a^ N (%)		Physical IPV^a^ N (%)	
	N^b^	%	No	Yes	No	Yes
**Any pathology** (N = 718)	539	75.1	417 (73)	122 (81)	514 (74)	25 (96)*
**Anemia**	304	39.3	216 (38)	67 (45)	271 (39)	12 (48)
**Urinary tract infection**	195	25.3	127 (23)	56 (37)*	173 (25)	10 (39)
**Vaginal bleeding**	125	16.0	88 (16)	21 (14)	105 (15)	4 (15)
**Gestational diabetes**	62	8.0	43 (8)	10 (7)	52 (8)	1 (4)
**Spontaneous preterm labour**	55	7.1	32 (6)	19 (13)*	47 (7)	4 (15)
**Vaginal infection**	53	6.9	30 (5)	20 (13)*	47 (7)	3 (12)
**Gestational hypertension**	51	6.6	39 (7)	7 (5)	45 (7)	1 (4)
**Others pathologies**	116	14.9	-	-	-	-
**Emergencies** (N = 777)						
No	313	40.3	236 (42)	55 (36)	280 (41)	11 (42)
1	235	30.2	184 (32)	41 (27)*	217 (31)	8 (31)
>1	229	29.5	146 (26)	55 (36)*	194 (28)	7 (27)
**Antenatal hospitalization** (N = 776)						
No	616	79.4	458 (81)	113 (75)	556 (81)	15 (58)
1	100	12.9	72 (13)	18 (12)*	86 (12)	4 (15)*
>1	60	7.7	35 (6)	20 (13)*	48 (7)	7 (27)*

^a^IPV: Intimate partner violence. Prevalence of: IPV 21.3% (n = 153); psychological IPV 21% (n = 151); physical IPV (n = 26); both psychological and physical IPV (n = 24)

^b^N total = 779 women

* p<0.05

## Discussion

In this study, psychological IPV, reported by 1 in 5 mothers, was associated with urinary tract infection, vaginal infection and spontaneous preterm labour, and physical IPV, reported by 1 in 27 mothers, was associated with antenatal hospitalizations. As mothers with IPV have higher odds of complications, clinicians should be vigilant about the risk of IPV in pregnancy.

The strength of our investigation is that it was a population-based study focusing on capturing psychological violence with a validated tool. The sample provided data with a high (>90%) response rate. However, the small numbers, refusal to fill out questionnaires should always be methodological considerations. Empirically, the low proportion (<5%) of lost data should reassure about a minimum or non-existent effect on the validity of our results [8]. Another strength of the current study is the use of local language in the instrument to identify IPV amongst pregnant women and the training of midwives for data collection. One limitation of the study is that IPV was assessed during the immediate postpartum period, when women tend to feel particularly vulnerable and violence may have underreported [24]. Some women may be willing to report psychological, but not physical abuse, even when physical abuse has been experienced. Some may fear outcomes of disclosure, stigma, discrimination, shame, removal of other children in the home, etc. A further strength is that in-depth analyses showed that socio-demographic characteristics had no effect on outcome in the adjusted multivariate models (S1–S3 Tables).

Facts indicate that IPV during pregnancy is more common than others conditions routinely tested for in antenatal care [1,4,25]. It is increasingly being recognised that IPV that occurs in pregnancy can have devastating consequences for both the mother and her unborn baby [26]. The most direct consequences of IPV during pregnancy are injuries from physical assaults [27], resulting in extreme cases in the death of the mother or the foetus [21,28,29]. But focus on physical IPV only in pregnancy [27,30–35] defines the problem too narrowly for the victims and the unborn offspring. Moreover, psychological abuse reports may at times be markers for comorbid physical abuse or for later physical IPV risk. Psychological IPV in pregnancy, so far a largely overlooked area of research [12,13], has detrimental consequences for reproductive health [14].

We documented an association between psychological IPV during pregnancy and spontaneous preterm labour, like in other studies of physical IPV [27,32,36]. Also, interestingly, we found that urinary tract infection and vaginal infections were associated with psychological IPV during pregnancy. Typically, these infections are commonly associated with sexual [37] or physical violence [27,32,34]. Another study [38] found that women victims of psychological or physical and sexual violence were at greater risk of infections, but this relationship was confounded by the level of education, social class, type of union or ethnic group. In our study, the socio-demographic characteristics (type of relationship and cohabitation) did not influence to relations observed in the adjusted models.

Our study also found that physical IPV raised the odds of antenatal hospitalized after adjustment for others confounders. Antenatal hospitalization has rarely been evaluated in IPV studies, except in studies of particularly high IPV prevalence [36] or where abused women were identified by police reports [39]. Women with lack of kin support were at increased risk of spontaneous preterm labour. Lack of kin support may also be a risk factor of psychological and physical IPV violence during pregnancy [8].

The generalization of our observations to other samples of pregnant women should pay attention to some issues concerning the model of care in our sample. For instance, women who present for an ultrasound, but no other antenatal care, may differ in risk of IPV compared to those who present regularly for antenatal care. The findings may or may not generalize to women living in other countries, where access to prenatal care is more or less available than in Spain. Moreover, as the data are now almost 10 years old, having been collected as part of an extended national project, there may be implications for current cohorts with respect how our results should be incorporated in current policy and practice. These issues are relevant in examining applicability of our findings.

A range of mechanisms may be proposed to explain the association between IPV and maternal outcomes [40]. A direct pathway in cases of physical and sexual trauma is self-evident. Another pathway is linked to the effect of the stress produced by IPV during pregnancy. Stress may exacerbate pre-existing conditions such as chronic hypertension, may lead to pregnancy complications such as preterm labour, may affect the reproductive endocrine system, reflect in unhealthy behaviours such as alcohol or drug use during pregnancy and affect maternal mental health [30,32,40]. Future research should explore the causal biological pathways of IPV on maternal and offspring outcomes.

## Conclusions

Experience of IPV during pregnancy affects maternal health, with psychological IPV playing a recognisable role. Mothers with IPV are deeply concerned about the risk of harm to the unborn baby. Their desire to find ways out of this predicament is fraught with difficulties and often goes unsupported [41]. Obstetricians, gynaecologists, midwives and other allied health care professionals must act as active screeners to identify IPV, particularly of the undervalued psychological IPV. Early detection of IPV must be followed by proper multidisciplinary input to protect the victims [42].

## Supporting information

S1 File Index of Spouse Abuse (ISA) instrument. (DOCX)

S1 Table Univariate and multivariate regression models for spontaneous preterm labour. (DOCX)

S2 Table Univariate and multivariate regression models for urinary tract infection and vaginal infection during pregnancy. (DOCX)

S3 Table Univariate and multivariate regression models for attendance to emergency services and antenatal hospitalization. (DOCX)
