# Supplementary material for: Maternal outcomes associated to psychological and physical intimate partner violence during pregnancy: A cohort study and multivariate analysis
Source: PLoS One. 2019 Jun 13;14(6):e0218255. doi: 10.1371/journal.pone.0218255 (PMC6564538; doi:10.1371/journal.pone.0218255)
Supplement: S1 File — (DOCX) [file pone.0218255.s001.docx]

INDEX OF SPOUSE ABUSE (ISA):

This questionnaire is designed to measure the degree of abuse you have experienced in your relationship with your partner. It is not a test, so there are no right or wrong answers. Answer each item as carefully and accurately as can by placing a number beside each one as follows:

1 Never

2 Rarely

3 Occasionally

4 Frequently

5 Very Frequently

Please begin.

1. My partner belittles me. (1)

2. My partner demands obedience to his whims. (17)

3. My partner becomes surly and angry if I tell him he is drinking too much. (15)

4. My partner makes me perform sex acts that I do not enjoy or like. (50)

5. My partner becomes very upset if dinner, housework or laundry is not done when he thinks it should be. (4)

6. My partner is jealous and suspicious of my friends. (8)

7. My partner punches me with his fists. (75)

8. My partner tells me I am ugly and unattractive. (26)

9. My partner tells me I really couldn't manage or take care of myself without him. (8)

10. My partner acts like I am his personal servant. (20)

11. My partner insults or shames me in front of others. (41)

12. My partner becomes very angry if I disagree with his point of view. (15)

13. My partner threatens me with a weapon. (82)

14. My partner is stingy in giving me enough money to run our home. (12)

15. My partner belittles me intellectually. (20)

16. My partner demands that I stay home to take care of the children. (14)

17. My partner beats me so badly that I must seek medical help. (98)

18. My partner feels that I should not work or go to school. (21)

19. My partner is not a kind person. (13)

20. My partner does not want me to socialize with my female friends. (18)

21. My partner demands sex whether I want it or not. (52)

22. My partner screams and yells at me. (38)

23. My partner slaps me around my face and head. (80)

24. My partner becomes abusive when he drinks. (65)

25. My partner orders me around. (29)

26. My partner has no respect for my feelings. (39)

27. My partner acts like a bully towards me. (44)

28. My partner frightens me. (55)

29. My partner treats me like a dunce. (29)

30. My partner acts like he would like to kill me. (80)

P (Physical): 3, 4, 7, 13, 17, 22-24, 27, 28, 30.

NP (Non Physical): 1, 2, 5, 6, 8-12, 14-16, 18-21, 25, 26, 29.

Source: Hudson WW, Mclntosh SR. The assessment of spouse abuse: two quantifiable dimensions. J Marriage Fam. 1981;43:873-85.

The rationale of the exposure measurement is that each of the ISA items represents some form of behaviour or partner interaction that is considered to be abusive (S1 File). However, some of these behaviours represent very serious types of abuse and others are less serious. Because the ISA items represent various degrees of abuse, these differences must be taken into account in the scoring and interpretation of the item responses. Since the ISA was designed to measure two different types of abuse, it is necessary to compute two different scores for each respondent: an ISA-P score that represents the severity of physical abuse and an ISA-NP score that represents the severity of nonphysical or psychological abuse. Both the ISA-P and ISA-NP scores range from 0 to 100 where a low score indicates the relative absence of abuse and the higher scores represent the presence of a greater degree or amount of abuse. There is a number in parentheses at the end of each item (S1 File). These numbers are item weights that are used in scoring the ISA to account for the fact that some of the items represent very serious forms of abuse while others do not. Although the ISA subscales appear to have excellent content, discriminant, construct, and factorial validity, the clinical utility of the ISA will be greatly improved if it is possible to establish valid cutting scores for the two subscale. In order to establish such clinical cutting scores for the ISA subscales, separate cumulative frequency distributions were prepared for the clinical criterion groups with respect to both the ISA-P and ISA-NP scores. The results showed that an ISA-P score of 10 and an ISA-NP score of 25 are the best clinical cutting scores in terms of their ability to minimize the sum of false positives and false negatives. Since the use of differential item weights does not produce lower validity coefficients as compared to the use of equal item weights, strict adherence to the scoring procedures is strongly recommended.
