# Supplementary material for: Maternal outcomes associated to psychological and physical intimate partner violence during pregnancy: A cohort study and multivariate analysis
Source: PLoS One. 2019 Jun 13;14(6):e0218255. doi: 10.1371/journal.pone.0218255 (PMC6564538; doi:10.1371/journal.pone.0218255)
Supplement: S1 Table — (DOCX) [file pone.0218255.s002.docx]

| **S1 Table. Univariate and multivariate regression models for spontaneous preterm labour.** | | | | |
| --- | --- | --- | --- | --- |
|  | **Spontaneous preterm labour** | | | |
|  | **N** | **Fr (%)** | **COR (95% CI)** | **AOR (95% CI)** |
| **Psychological IPV** |  |  |  |  |
| No | 564 | 32 (6) | 1 | 1 |
| Yes | 151 | 19 (13) | 2.4 (1.3-4.4)* | 2.2 (1.1-4.5)* |
| **Physical IPV** |  |  |  |  |
| No | 689 | 47 (7) | 1 | 1 |
| Yes | 26 | 4 (15) | 2.5 (0.8-7.5) | 0.7 (0.3-3.3) |
| **Age (years)** |  |  |  |  |
| <20 | 31 | 2 (6) | 1 | 1 |
| 20-24 | 101 | 8 (8) | 1.3 (0.3-6.2) | 0.7 (0.1-5.0) |
| 25-29 | 198 | 14 (7) | 1.1 (0.2-5.1) | 0.7 (0.1-4.6) |
| 30-34 | 275 | 25 (9) | 1.5 (0.3-6.4) | 1.5 (0.2-9.7) |
| 35-39 | 119 | 6 (5) | 0.8 (0.2-4.0) | 0.7 (0.1-5.4) |
| ≥ 40 | 31 | 0 | empty | empty |
| **Relationship** |  |  |  |  |
| Married | 497 | 31 (6) | 1 | 1 |
| Committed | 106 | 7 (7) | 1.1 (0.5-2.5) | 1.1 (0.4-3.0) |
| Non- committed | 170 | 17 (10) | 1.7 (0.9-3.1) | 1.6 (0.7-3.9) |
| **Schooling (years)** |  |  |  |  |
| <7 | 292 | 16 (6) | 1 |  |
| 7 - 12 | 376 | 34 (9) | 1.7 (0.9-3.2) | 1.6 (0.8-3.4) |
| >12 | 106 | 5 (5) | 0.9 (0.3-2.4) | 0.8 (0.2-2.8) |
| **Employment** |  |  |  |  |
| Housewife | 170 | 12 (7) | 1 | 1 |
| Unemployed | 162 | 15 (9) | 1.3 (0.6-3.0) | 1.4 (0.6-3.4) |
| Employed | 427 | 26 (6) | 0.9 (0.4-1.7) | 0.9 (0.4-2.1) |
| Student | 15 | 2 (13) | 2.0 (0.4-10.0) | 1.7 (0.1-20.9) |
| **Nacionality** |  |  |  |  |
| Spanish | 707 | 49 (7) | 1 | 1 |
| Other | 68 | 6 (9) | 1.3 (0.5-3.1) | 0.9 (0.3-2.8) |
| **Cohabitation** |  |  |  |  |
| Partner | 705 | 51 (7) | 1 | 1 |
| Others | 70 | 4 (6) | 0.8 (0.3-2.2) | 0.2 (0.0-1.3) |
| **Kin support** |  |  |  |  |
| Yes | 735 | 47 (6) | 1 | 1 |
| No | 37 | 8 (22) | 4.0 (1.8-9.3)* | 4.7 (1.7-12.8)* |
| IPV = Intimate partner violence; COR = crude odds ratio; AOR = adjusted odds ratio  * Significant 95% CI (does not include COR or AOR null value) | | | | |
