# Supplementary material for: Maternal outcomes associated to psychological and physical intimate partner violence during pregnancy: A cohort study and multivariate analysis
Source: PLoS One. 2019 Jun 13;14(6):e0218255. doi: 10.1371/journal.pone.0218255 (PMC6564538; doi:10.1371/journal.pone.0218255)
Supplement: S2 Table — (DOCX) [file pone.0218255.s003.docx]

| **S2 Table. Univariate and multivariate regression models for urinary tract infection and vaginal infection during pregnancy.** | | | | | | | | |
| --- | --- | --- | --- | --- | --- | --- | --- | --- |
|  | **Urinary tract infection** | | | | **Vaginal infection** | | | |
|  | **N** | **Fr (%)** | **COR (95% CI)** | **AOR (95% CI)** |  | **Fr (%)** | **COR (95% CI)** | **AOR (95% CI)** |
| **Psychological IPV** |  |  |  |  |  |  |  |  |
| No | 562 | 127 (23) | 1 | 1 |  | 30 (5) | 1 | 1 |
| Yes | 150 | 56 (37) | 2.0 (1.4-3.0)* | 1.9 (1.2-3.0)* |  | 20 (13) | 2.7 (1.5-5.0)* | 2.4 (1.2-4.7)* |
| **Physical IPV** |  |  |  |  |  |  |  |  |
| No | 686 | 173 (25) | 1 | 1 |  | 47 (7) | 1 | 1 |
| Yes | 26 | 10 (39) | 1.9 (0.8-4.2) | 1.2 (0.5-3.1) |  | 3 (12) | 1.8 (0.5-6.1) | 0.6 (0.1-2.5) |
| **Age (years)** |  |  |  |  |  |  |  |  |
| <20 | 30 | 12 (40) | 1 | 1 |  | 0 | 1 | 1 |
| 20-24 | 101 | 40 (40) | 1.0 (0.4-2.3) | 1.3 (0.5-3.5) |  | 9 (9) | 2.9 (0.4-24.1) | 1.0 (0.1-9.3) |
| 25-29 | 196 | 42 (21) | 0.4 (0.2-0.9)* | 0.6 (0.2-1.7) |  | 21 (11) | 3.6 (0.5-27.8) | 2.1 (0.3-17.9) |
| 30-34 | 273 | 68 (25) | 0.5 (0.2-1.1) | 0.8 (0.3-2.2) |  | 14 (5) | 1.6 (0.2-12.8) | 1.3 (0.2-11.1) |
| 35-39 | 120 | 26 (22) | 0.4 (0.2-0.9)* | 0.7 (0.2-2.1) |  | 7 (6) | 1.9 (0.2-15.7) | 1.9 (0.2-17.5) |
| ≥ 40 | 31 | 4 (13) | 0.2 (0.1-0.8)* | 0.3 (0.1-1.4) |  | 1 (3) | 1 (omitted) | 1 (omitted) |
| **Relationship** |  |  |  |  |  |  |  |  |
| Married | 495 | 109 (22) | 1 | 1 |  | 24 (5) | 1 | 1 |
| Committed | 104 | 24 (23) | 1.1 (0.6 -1.8) | 0.8 (0.5-1.5) |  | 10 (10) | 2.1 (1.0-4.5)* | 1.8 (0.8-4.2) |
| Non- committed | 170 | 62 (37) | 2.0 (1.4-3.0)* | 1.6 (0.9-2.6) |  | 19 (11) | 2.5 (1.3-4.6)* | 2.1 (0.9-4.7) |
| **Schooling (years)** |  |  |  |  |  |  |  |  |
| <7 | 288 | 84 (29) | 1 | 1 |  | 22 (8) | 1 | 1 |
| 7 - 12 | 376 | 94 (25) | 0.8 (0.6-1.1) | 1.0 (0.6-1.4) |  | 27 (7) | 0.9 (0.5-1.7) | 1.1 (0.5-2.2) |
| >12 | 106 | 17 (16) | 0.5 (0.3-0.8)* | 0.6 (0.3-1.1) |  | 4 (4) | 0.5 (0.2-1.4) | 0.8 (0.3-2.8) |
| **Employment** |  |  |  |  |  |  |  |  |
| Housewife | 168 | 47 (28) | 1 | 1 |  | 11 (7) | 1 | 1 |
| Unemployed | 161 | 48 (30) | 1.1 (0.7-1.8) | 1.1 (0.6-1.8) |  | 18 (11) | 1.8 (0.8-3.9) | 1.8 (0.8-4.3) |
| Employed | 427 | 94 (22) | 0.7 (0.5-1.1) | 0.8 (0.5-1.2) |  | 23 (5) | 0.8 (0.4-1.7) | 0.9 (0.4-2.1) |
| Student | 15 | 6 (40) | 1.7 (0.6-5.1) | 0.8 (0.2-3.4) |  | 1 (7) | 1.0 (0.1-8.5) | 2.7 (0.2-31.7) |
| **Nacionality** |  |  |  |  |  |  |  |  |
| Spanish | 705 | 179 (25) | 1 | 1 |  | 45 (5) | 1 | 1 |
| Other | 66 | 16 (24) | 0.9 (0.5-1.7) | 0.7 (0.3-1.3) |  | 8 (12) | 2.0 (0.9-4.5) | 1.6 (0.6-4.0) |
| **Cohabitation** |  |  |  |  |  |  |  |  |
| Partner | 701 | 169 (24) |  | 1 |  | 46 (7) | 1 | 1 |
| Others | 70 | 26 (37) | 1.9 (1.1-3.1)* | 1.0 (0.5-2.1) |  | 7 (10) | 1.6 (0.7-3.7) | 1.2 (0.4-3.7) |
| **Kin support** |  |  |  |  |  |  |  |  |
| Yes | 731 | 183 (25) | 1 | 1 |  | 49 (7) | 1 | 1 |
| No | 37 | 12 (32) | 1.4 (0.7-2.9) | 1.2 (0.5-2.7) |  | 4 (11) | 1.7 (0.6-5.0) | 0.8 (0.2-2.9) |
| IPV = Intimate partner violence; COR = crude odds ratio; AOR = adjusted odds ratio  * Significant 95% CI (does not include COR or AOR null value) | | | | | | | | |
