# Supplementary material for: Maternal outcomes associated to psychological and physical intimate partner violence during pregnancy: A cohort study and multivariate analysis
Source: PLoS One. 2019 Jun 13;14(6):e0218255. doi: 10.1371/journal.pone.0218255 (PMC6564538; doi:10.1371/journal.pone.0218255)
Supplement: S3 Table — (DOCX) [file pone.0218255.s004.docx]

| **S3 Table. Univariate and multivariate regression models for attendance to emergency services and antenatal hospitalization.** | | | | | | | | |
| --- | --- | --- | --- | --- | --- | --- | --- | --- |
|  | **Emergencies** | | | | **Antenatal Hospitalization** | | | |
|  | **N** | **Fr (%)** | **COR (95% CI)** | **AOR (95% CI)** | **N** | **Fr (%)** | **COR (95% CI)** | **AOR (95% CI)** |
| **Psychological IPV** |  |  |  |  |  |  |  |  |
| No | 566 | 330 (58) | 1 | 1 | 565 | 107 (19) | 1 | 1 |
| Yes | 151 | 96 (64) | 1.2 (0.9-1.8) | 1.1 (0.7-1.8) | 151 | 38 (25) | 1.4 (0.9-2.2) | 1.0 (0.6-1.8) |
| **Physical IPV** |  |  |  |  |  |  |  |  |
| No | 691 | 411 (60) | 1 | 1 | 690 | 134 (19) | 1 | 1 |
| Yes | 26 | 15 (58) | 0.9 (0.4-2.0) | 0.7 (0.3-1.9) | 26 | 11 (42) | 3.0 (1.4-6.8)* | 2.6 (1.1-7.1)* |
| **Age (years)** |  |  |  |  |  |  |  |  |
| <20 | 31 | 14 (45) | 1 | 1 | 31 | 5 (16) | 1 | 1 |
| 20-24 | 101 | 74 (73) | 3.3 (1.5-7.7)* | 3.7 (1.3-10.0)* | 100 | 33 (33) | 2.6 (1.0-7.3)* | 3.8 (0.9-15.2) |
| 25-29 | 199 | 126 (63) | 2.1 (1.0-4.5)* | 2.5 (0.9-6.9) | 199 | 51 (26) | 1.8 (0.6-4.9) | 2.5 (0.6-10.2) |
| 30-34 | 275 | 157 (57) | 1.6 (0.8-3.4) | 2.0 (0.7-5.5) | 275 | 44 (16) | 1.0 (0.4-2.7) | 1.5 (0.3-6.3) |
| 35-39 | 120 | 69 (58) | 1.6 (0.7-3.6) | 2.4 (0.8-7.0) | 120 | 19 (16) | 1.0 (0.3-2.9) | 1.3 (0.3-5.8) |
| ≥ 40 | 31 | 14 (45) | 1.0 (0.4-2.7) | 1.2 (0.3-4.5) | 31 | 4 (13) | 0.8 (0.2-3.2) | 1.0 (0.2-6.4) |
| **Relationship** |  |  |  |  |  |  |  |  |
| Married | 498 | 280 (56) | 1 | 1 | 498 | 100 (20) | 1 | 1 |
| Committed | 106 | 68 (64) | 1.4 (0.9-2.1) | 1.1 (0.7-1.9) | 105 | 15 (14) | 0.7 (0.4-1.2) | 0.5 (0.2-1.0) |
| Non- committed | 171 | 115 (67) | 1.6 (1.1-2.3)* | 1.4 (0.8-2.3) | 171 | 45 (26) | 1.4 (1.0-2.1) * | 1.1 (0.6-1.9) |
| **Schooling (years)** |  |  |  |  |  |  |  |  |
| <7 | 293 | 188 (64) | 1 | 1 | 292 | 74 (25) | 1 | 1 |
| 7 - 12 | 377 | 228 (61) | 0.9 (0.6-1.2) | 0.9 (0.6-1.3) | 377 | 81 (22) | 0.8 (0.6-1.2) | 0.8 (0.5-1.3) |
| >12 | 106 | 48 (45) | 0.5 (0.3-0.7)* | 0.5 (0.3-0.9)* | 106 | 4 (4) | 0.1 (0.0-0.3)* | 0.1 (0.0-0.5)* |
| **Employment** |  |  |  |  |  |  |  |  |
| Housewife | 169 | 96 (57) | 1 | 1 | 168 | 45 (27) | 1 | 1 |
| Unemployed | 162 | 108 (67) | 1.5 (0.9-2.4) | 1.5 (0.9-2.5) | 162 | 46 (28) | 1.1 (0.7-1.8) | 1.2 (0.7-2.2) |
| Employed | 430 | 252 (59) | 1.1 (0.8-1.5) | 1.2 (0.8-1.9) | 430 | 66 (15) | 0.5 (0.3-0.8)* | 0.7 (0.4-1.1) |
| Student | 15 | 7 (47) | 0.7 (0.2-1.9) | 0.8 (0.2-3.1) | 15 | 3 (20) | 0.7 (0.2-2.5) | 1.0 (0.2-5.7) |
| **Nacionality** |  |  |  |  |  |  |  |  |
| Spanish | 708 | 427 (60) | 1 | 1 | 708 | 410 (20) | 1 | 1 |
| Other | 68 | 37 (54) | 1.2 (0.8-2.1) | 1.6 (0.9-3.0) | 68 | 20 (29) | 1.7 (0.9-2.9) | 1.0 (0.5-2.0) |
| **Cohabitation** |  |  |  |  |  |  |  |  |
| Partner | 706 | 421 (60) | 1 | 1 | 705 | 144 (20) | 1 | 1 |
| Others | 71 | 43 (61) | 1.0 (0.6-1.7) | 0.9 (0.4-1.8) | 71 | 16 (23) | 1.1 (0.6-2.0) | 0.7 (0.3-1.6) |
| **Kin support** |  |  |  |  |  |  |  |  |
| Yes | 737 | 438 (59) | 1 | 1 | 736 | 141 (20) | 1 | 1 |
| No | 37 | 24 (65) | 1.3 (0.6-2.5) | 1.2 (0.5-2.8) | 37 | 17 (46) | 3.6 (1.8-7.0)* | 2.2 (1.0-5.0) |
| **Any pathology** |  |  |  |  |  |  |  |  |
| No | 185 | 79 (43) | 1 | 1 | 185 | 24 (13) | 1 | 1 |
| Yes | 592 | 385 (65) | 2.5 (1.8-3.5)* | 2.6 (1.8-3.7)* | 591 | 136 (23) | 2.0 (1.2-3.2)* | 1.8 (1.1-3.1)* |
| IPV = Intimate partner violence; COR = crude odds ratio, AOR = adjusted odds ratio  * Significant 95% CI (does not include COR or AOR null value) | | | | | | | | |
